# Integration Analysis of Metabolome and Transcriptome Reveals the Effect of Lipopolysaccharide on Ovary Response to Stimulation

**DOI:** 10.1002/iid3.70309

**Published:** 2025-12-24

**Authors:** Xin Mu, Feng Zhu, Yudan Zhang, Mei‐Li Pei

**Affiliations:** ^1^ Clinacal Department The Assisted Reproductive Medicine Center, Northwest Women's and Children's Hospital Xi'an Shaanxi People's Republic of China; ^2^ Center for Translational Medicine The First Affiliated Hospital of Xi'an Jiaotong University Xi'an Shaanxi People's Republic of China; ^3^ Department of Gynecology and Obstetrics The First Affiliated Hospital of Xi'an Jiaotong University Xi'an Shaanxi People's Republic of China

**Keywords:** correlation analysis, LPS, metabolomics, transcriptomics

## Abstract

**Objective:**

This study aimed to elucidate the underlying mechanisms by which lipopolysaccharide (LPS) influences differential ovarian responses to controlled ovarian stimulation at the metabolite and gene levels.

**Materials and Methods:**

A total of 16 female mice were randomly allocated into LPS and control group. Each mice underwent controlled ovarian stimulation. Oocytes were gathered and quantified. Ovarian tissue samples from 8 mice and blood samples from 16 mice were collected. Metabolomics analysis was conducted for both ovary tissues and blood samples. Transcriptome sequencing analysis was used for ovary tissues. Spearman's correlation analysis was performed to identify the metabolites and their corresponding differential transcripts genes within the key signaling pathway.

**Results:**

The number (86 vs. 122) and rate (49.7% vs. 78.2%) of metaphase II (MII) oocytes were markedly lower in LPS group (*p* < 0.001). Compared with control group, metabolites related to carbohydrates and lipid were decreased in ovary tissue of LPS group while metabolites related to amino acid and fatty acid beta‐oxidation were decreased in serum of LPS group. Within the category of biological processes, terms associated with the regulation of lipid metabolic process and steroid metabolic, biosynthetic process were downregulated in the LPS group. Correlation analysis indicated five genes (Lcn2, S100a9, S100a8, Muc5b, Muc5ac) in IL‐17 signaling pathway were positively related to the catabolites of polyunsaturated fatty acids and negatively related to dihydroxyacetone phosphate.

**Conclusions:**

This study provided evidence that inflammation has damaging effect on ovary response to controlled ovarian stimulation, linked to dysfunctions in lipid, carbohydrate, and amino acid metabolism. IL‐17 signal pathways may play a critical role in this process.

## Graphical abstract

This study provided evidence of inflammation on damaging ovary response to controlled ovarian stimulation, linked to dysfunctions in lipid, carbohydrate, and amino acid metabolism. IL‐17 signal pathways may play a critical role in this process.

## 1 Introduction

Controlled ovarian stimulation (COS) represents a critical component of the in‐vitro fertilization (IVF) protocol, significantly contributing to its success rate. The correlation between an appropriate ovarian response to COS and the probability of live birth rate is well‐documented [1]. Despite considerable advancements in COS protocols, a fraction of individuals fails to achieve a satisfactory response to stimulation, a condition referred to as poor ovarian response (POR). This condition affects ~10%–40% of patients undergoing IVF treatment [2]. In Italy, it was reported that 6.7% of IVF cycles were prematurely terminated due to POR [3]. Factors such as age, body mass index, genetic traits, environmental pollution, and oxidative stress have been linked to POR [3, 4, 5, 6]. Nevertheless, the underlying pathological mechanisms of POR remain largely unexplored.

Inflammation serves as the body's defense mechanism against harmful and foreign stimuli. Inflammation, particularly asymptomatic inflammation, has been detected in some women experiencing infertility. Accumulating evidence suggests that increased inflammation may be linked to various reproductive diseases, including obesity, polycystic ovary syndrome, compromised implantation, and embryo development [7, 8], ultimately leading to infertility. Pro‐inflammatory immune responses may adversely affect the ovarian response. From a molecular perspective, elevated levels of interleukins, specifically IL‐15, in follicle fluid have been associated with smaller follicles and immature oocytes [9]. A study conducted by Wu et al. revealed that the levels of peripheral blood CD56+ natural killer (NK) cells, NK cytotoxic activity, CD19+ B cells, and the Th1/Th2 cell ratio were significantly elevated in the POR group [10]. However, the precise mechanism through which inflammation impairs the ovarian response remains to be fully elucidated.

Lipopolysaccharide (LPS), an endotoxin synthesized by gram‐negative bacteria, is identified by its specific receptor present on immune cells, which triggers the discharge of proinflammatory cytokines [11]. In mammalian studies, LPS has been extensively employed to create a proinflammatory model. Existing literature suggests that an elevated circulating concentration of LPS may result in fertility impairment by suppressing steroid secretion and affecting the function of granulosa cells [12, 13]. Furthermore, LPS can instigate ovarian inflammation, fibrosis, and granulosa cell apoptosis, thereby establishing a primary ovarian insufficiency model in mice [14].

In the present study, we employed LPS to establish a proinflammatory model in mice. We performed an integrated analysis of metabolomics and transcriptomics with the aim of elucidating the potential mechanism underlying the effect of LPS on differential ovarian response at the metabolite and gene levels.

## 2 Materials and Methods

### 2.1 Animal Management and Treatment

The animal study was performed in the institutional animal facility at the First Affiliated Hospital of Xi'an Jiaotong University and approved by the Health Science Center of XJTU Approval for Research Involving Animals (XJTUAE2024‐1872). All experimental procedures were conducted in accordance with the Guide for the Care and Use of Laboratory Animals of Xi'an Jiaotong University. Sixteen female C57 mice, aged 5–6 weeks, were procured from Beijing Vital River Laboratory Animal Technology Co. Ltd. (Beijing, China). They were accommodated under a 12‐h light/dark cycle in a controlled temperature between 21°C and 22°C, and 20%–30% humidity environment. The animals had unrestricted access to food and water.

Following a week of acclimatization, the mice were randomly assigned to two experimental groups: eight in the LPS group and eight in the control group. Based on prior studies [15], mice in the LPS group were administered intraperitoneal injections of LPS (0.1 mg/kg; L3024, Sigma‐Aldrich, St. Louis, MO, USA) once daily for 3 consecutive days to establish a systemic inflammatory model. Twenty‐four hours following the final LPS administration, mice were intraperitoneally injected with 10 IU of pregnant mare serum gonadotropin. After 48 h, a second intraperitoneal injection of 10 IU human chorionic gonadotropin (hCG) was administered between 7:00 and 8:00 p.m. to induce a COS model. In the control group, mice were similarly administered intraperitoneal injections of 0.1 mg/kg saline once daily over 3 consecutive days. Thirteen hours post‐hCG treatment (between 8 and 9 a.m.), the mice were euthanized under anesthesia according to ethical research guidelines. Ovulated cumulus–oocyte complexes (COCs) were then isolated from the ampullary region of the oviducts. The COCs were washed three times in M2 medium supplemented with 0.5 mg/mL hyaluronidase to remove surrounding cumulus cells. Denuded oocytes were subsequently collected and counted using a stereomicroscope. Oocytes exhibiting a clearly visible metaphase plate and the presence of the first polar body were classified as metaphase II (MII) oocytes. Ovarian tissue samples from 8 mice (4/group) and blood samples from 16 mice (8/group) were collected and stored at −80°C until further processing for RNA sequencing (RNA‐seq) and metabolomics analyses.

### 2.2 Transcriptome Sequencing Analysis

Ovarian total RNA was isolated using TRIzol reagent (Invitrogen, USA) in accordance with the manufacturer's guidelines. Library preparation and high‐throughput sequencing were carried out on the Illumina NovaSeq 6000 system by Nanjing Personal Biotechnology Co. Ltd. The reference genome and associated gene annotations were retrieved from a public database. Clean reads were subsequently aligned to the reference genome using HISAT2 (version 2.0.5). Gene expression abundance was quantified using HTSeq (0.9.1) and DESeq (1.39.0). Genes with a log fold higher than 1 and a adjusted *p* value < 0.05 were identified as differentially expressed genes (DEGs). All genes were annotated with Gene Ontology (GO) terms, and the distribution of DEGs across each GO category was quantified. GO functional enrichment was performed using the topGO R package (v2.40.0), applying a significance threshold of *p* <  0.05. Kyoto Encyclopedia of Genes and Genomes (KEGG) pathway enrichment was analyzed with ClusterProfiler (v3.16.1), and significantly enriched pathways were defined by the same cutoff.

### 2.3 Metabolites Extraction and Metabolomics Profiling

Metabolites extraction from ovarian tissues (four per group) was conducted in accordance with standardized protocols. Accurately weighed samples (25 ± 1 mg) were placed in tubes with ceramic beads and combined with 500 μL of extraction solvent. Following a 30‐s vortex, the mixture underwent mechanical homogenization at 35 Hz for 4 min, succeeded by ultrasound treatment for 5 min in a 4°C water bath. This sequence was executed three times to ensure efficient disruption and extraction. The resulting suspensions were subsequently maintained at −40°C for 1 h to promote metabolite precipitation. After incubation, the samples were clarified by centrifugation at 12,000 rpm for 15 min at 4°C, and the resulting supernatants were transferred into clean glass vials for metabolomic profiling. Serum sample preparation followed a comparable yet scaled protocol. A volume of 100 μL serum was mixed with 400 μL extraction buffer, subjected to vortexing (30 s), and then ultrasonicated at 4°C for 10 min. After a 1‐h cooling step at −40°C to enhance protein precipitation, the mixture was centrifuged under identical parameters. The upper phase was carefully collected into fresh vials for subsequent instrumental analysis. A pooled quality control sample was assembled by combining equal aliquots from all experimental supernatants to assess analytical stability and reproducibility. Metabolic profiling was performed using a Vanquish UHPLC platform (Thermo Fisher Scientific, USA), paired with a Waters ACQUITY UPLC BEH Amide column (2.1 mm × 50 mm, 1.7 μm), and interfaced with an Orbitrap Exploris 120 high‐resolution mass spectrometer (Thermo Fisher Scientific). Full‐scan acquisition was managed via the system's native software. Post‐acquisition, peak data—comprising feature identifiers, sample labels, and normalized signal intensities—were processed using SIMCA version 16.0.2 (Sartorius Stedim Data Analytics AB, Umeå, Sweden). To evaluate inherent clustering and trends, unsupervised principal component analysis (PCA) was applied. Subsequently, supervised orthogonal partial least squares discriminant analysis (OPLS‐DA) was utilized to distinguish group‐dependent metabolic changes. Differential features were defined by variable importance in projection (VIP) scores exceeding 1 and statistical significance at *p* < 0.05 (Student's *t*‐test). Functional interpretation and pathway mapping were performed using curated bioinformatics resources including KEGG (https://www.genome.jp/kegg/) and MetaboAnalyst (https://www.metaboanalyst.ca/).

### 2.4 Combined of Metabolome and Transcriptome Analysis

The differentially expressed metabolites (DEMs) and differential transcripts were annotated in the KEGG database. Then the Spearman correlation analysis was performed to identify the metabolites and their corresponding differential transcripts genes within the key signaling pathway.

### 2.5 PPI Network and Functional Enrichment Analysis

Protein–protein interaction (PPI) network construction was performed via the STRING database (https://string-db.org/) using DEGs as input. A minimum required interaction score of 0.4 was applied as the confidence threshold. Topological characterization of the network revealed an average node degree of 1.85, a local clustering coefficient of 0.355, and a statistically significant PPI enrichment *p* value of < 1.0 × 10^−^^16^, indicating nonrandom associations among the input proteins. Functional annotation and enrichment analyses were carried out using STRING's integrated analytical tools. The resulting interaction networks and pathway maps were exported directly for visualization, without additional manual modifications.

### 2.6 qPCR Analysis

Quantitative real‐time PCR (qPCR) assays were carried out on Real‐Time PCR Detection System (Bio‐Rad, Hercules, CA, USA) following the manufacturer's recommendations. Complementary DNA (cDNA) was synthesized using the HiScript III RT SuperMix kit (Vazyme, Nanjing, China). Specific primers were synthesized by Sangon Biotech (Shanghai, China), and their sequences are provided in Supporting Information S4: Table 1.

### 2.7 Serum IL‐17 Analysis

Blood samples were collected from the postcaval vein and centrifuged at 4000 rpm for 10 min to isolate the serum. Serum IL‐17 were quantified using ELISA kits (MLbio, China) in accordance with the manufacturer's protocol.

### 2.8 Statistical Analysis

All statistical analyses were conducted using SPSS software (version 22.0; IBM Corp., Armonk, NY, USA). Continuous variables were evaluated using Student's *t*‐test, while categorical data were assessed via the chi‐square (*χ* ^2^) test. For omics‐level data, including differential gene expression and metabolite profiling, R software (version 1.0.8) was employed. In metabolomics analysis, features with a VIP score > 1 and a *p* value below 0.05 (derived from Student's *t*‐test) were considered to exhibit statistically significant alterations.

## 3 Results

### 3.1 Effect of LPS on Oocytes Number

**Figure 1:**
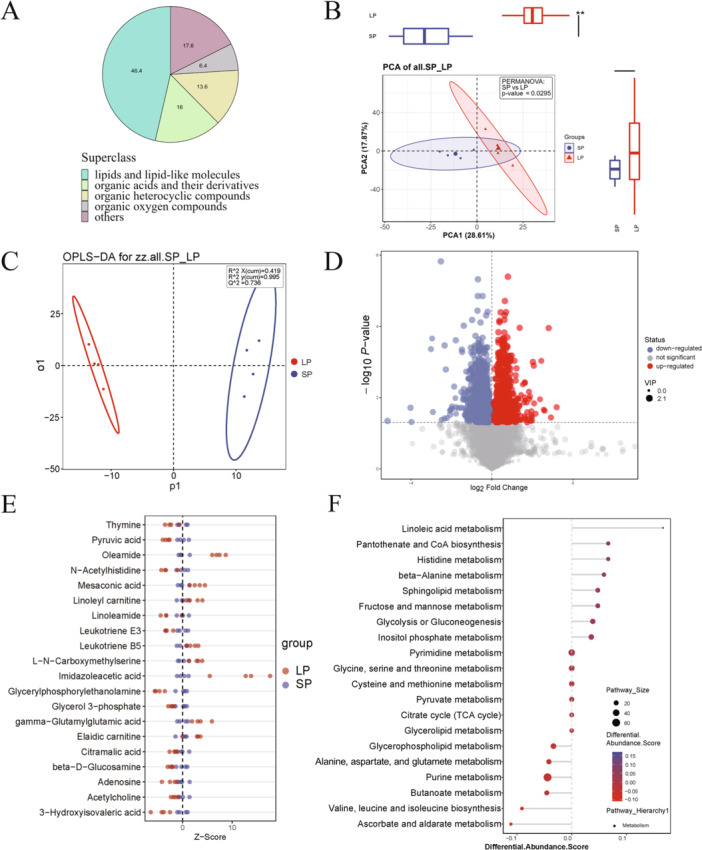
Metabolomic analysis of ovary tissue between control (SP) and LPS (LP) group. (A) Proportion of identified differentially expressed annotated metabolites in two groups. (B) Principal component analysis between two groups. (C) Orthogonal partial least squares discriminant analysis between two groups. (D) Volcano plots show patterns between two groups. The red dots represent the significantly upregulated metabolites and the blue dots represent the significantly downregulated metabolites. The gray dots represent nonsignificantly different metabolites. (E) *Z*‐score plot of 20 representative metabolites compared between two groups. (F) The 20 most significant KEGG pathways.

The serum IL‐17 levels were compared between the two groups. The results demonstrated that the IL‐17 concentration in the LPS‐treated group was significantly elevated compared to that in the control group (Supporting Information S1: Figure 1). Oocyte quantification was conducted using a somatic microscope. A significant decline in both the quantity and proportion of MII oocytes was identified in the LPS‐treated group. Specifically, 86 MII oocytes were retrieved from the LPS group, in contrast to 122 obtained from the control group. Correspondingly, the maturation rate was substantially lower in the LPS group (49.7%) compared to 78.2% observed in the controls (Table 1).

**Table 1:** Comparison of oocytes between LPS and control group.

	Control group	LPS group	*p*/*χ* ^2^
Total oocytes (*n*)	156	173	
MII numbers (*n*)	122	86	
Abnormal oocytes (*n*)	11	36	
Rate of MII (%)	78.2%	49.7%	**0.000**/28.644
Rate of abnormal oocytes (%)	7.1%	20.8%	**0.000**/12.680

*Note:* Statistically significant results (*p* < 0.05) are marked in bold.

Abbreviations: LPS, lipopolysaccharide; MII, metaphase II oocytes.

### 3.2 Metabolomic Differences Between Control and LPS Group

Liquid chromatography–tandem mass spectrometry was employed to characterize metabolic alterations induced by LPS exposure. In total, 125 DEMs were identified in ovarian tissues when comparing the LPS and control groups. Among them, the most enriched chemical classes included lipids and lipid‐like molecules (46.4%), organic acids and their derivatives (16%), organic heterocyclic compounds (13.6%), and organic oxygen compounds (6.4%) (Figure 1A). Unsupervised PCA demonstrated a distinct separation in metabolomic profiles between the two groups (Figure 1B), indicating considerable systemic differences. OPLS‐DA was subsequently performed to evaluate the model's robustness and classification performance, demonstrating robustness with R2Y (0.995) and Q2 (0.736) values (Figure 1C). The DEMs between the two groups were illustrated in a volcano plot, with 52 metabolites increased and 73 metabolites decreased in the LPS group based on univariate analysis (Figure 1D). Compared with control group, metabolites related to carbohydrates such as pyruvic acid and lipid such as glycerylphosphorylethanolamine and glycerol 3‐phosphate were decreased in LPS group (Figure 1E). Following the analysis of differential metabolites, the KEGG pathway database was employed to identify altered metabolic pathways. The most enriched pathways were purine metabolism, glycolysis or gluconeogenesis, fructose and mannose metabolism, and glycerophospholipid metabolism (Figure 1F).

**Figure 2:**
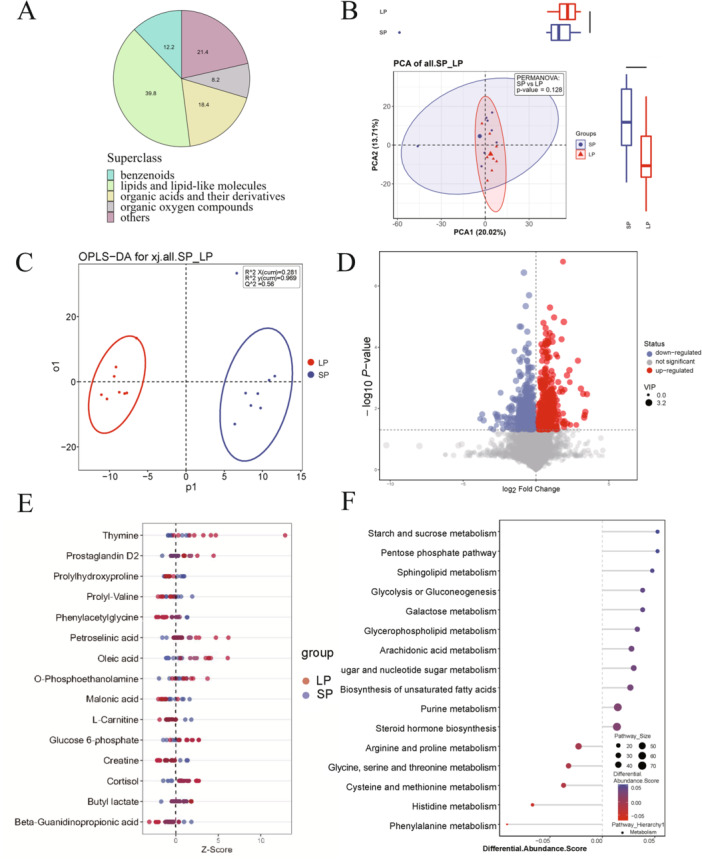
Metabolomic analysis of serum between control (SP) and LPS (LP) group. (A) proportion of identified differentially expressed annotated metabolites in two groups. (B) Principal component analysis between two groups. (C) Orthogonal partial least squares discriminant analysis between two groups. (D) Volcano plots show patterns between two groups. The red dots represent the significantly upregulated metabolites and the blue dots represent the significantly downregulated metabolites. The gray dots represent nonsignificantly different metabolites. (E) *Z*‐score plot of 15 representative metabolites compared between two groups. (F) The 16 most significant KEGG pathways in two groups.

For serum samples, 98 DEMs were annotated between the two groups. The top 5 categories of these metabolites were lipids and lipid‐like molecules (39.8%), organic acids and their derivatives (18.4%), benzenoids (12.2%), and organic oxygen compounds (8.2%) (Figure 2A). Unlike in ovarian tissue, PCA of the two serum groups did not demonstrate a clear separation, despite OPLS‐DA indicating good accuracy and validation (Figure 2B,C). Differential metabolite comparison revealed 56 increased and 42 decreased metabolites in the LPS group, as depicted in a volcano plot (Figure 2D). Compared with control group, metabolites related to amino acid such as prolylhydroxyproline, prolyl‐valine, and fatty acid beta‐oxidation such as l‐carnitine were decreased in LPS group (Figure 2E). The most enriched KEGG pathways were purine metabolism, steroid hormone metabolism, biosynthesis of unsaturated fatty acids, amino sugar, glycolysis or gluconeogenesis, and glycerophospholipid metabolism (Figure 2F).

**Figure 3:**
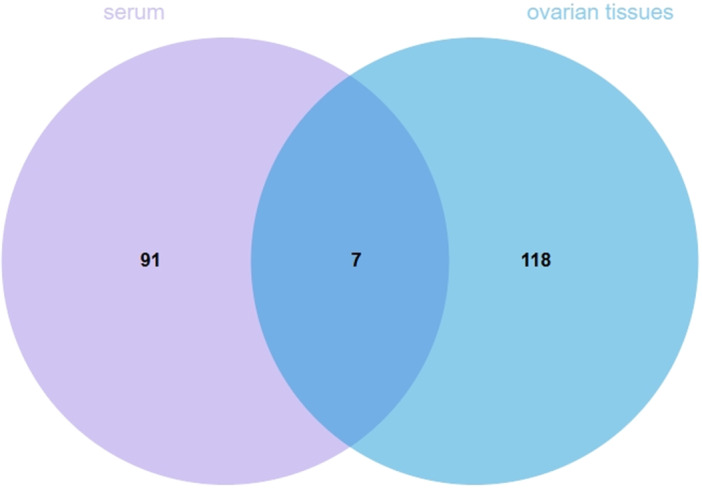
The Venn diagram showed common and unusual patterns at the superclass level between serum and ovarian tissues.

To identify shared and distinct metabolites between serum and ovarian tissues, Venn diagram analyses were performed. The analysis revealed seven co‐expressed differential metabolites at the superclass level, indicating a degree of metabolic similarity between the two tissue types (Figure 3).

### 3.3 Transcriptomic Differences Between Control and LPS Group

**Figure 4:**
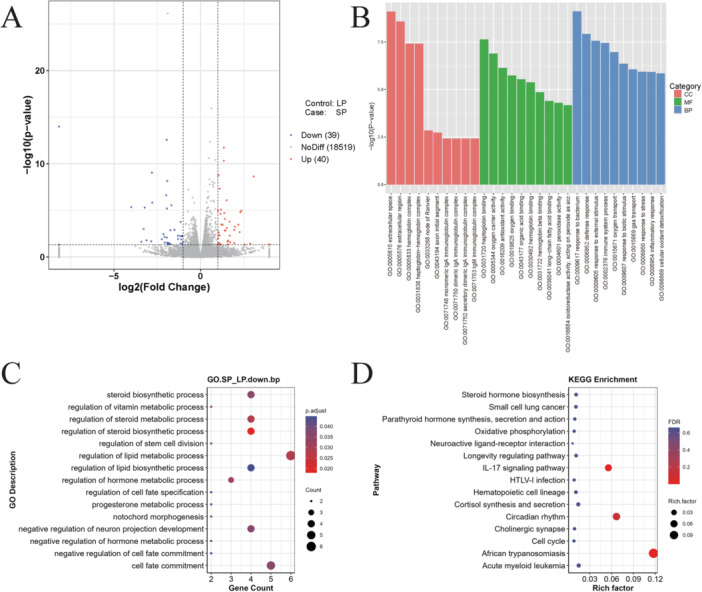
Transcriptomic analysis of ovary tissue between control (SP) and LPS (LP) group. (A) Volcano plots show different expressed genes between two groups. The red dots represent the significantly upregulated genes and the blue dots represent the significantly downregulated genes. The gray dots represent nonsignificantly different genes. (B) The top 10 enriched GO Gene Ontology terms in two groups. The red bar represents cellular component, the green bar represents molecular function and the blue bar represents biological process. (C) The significant downregulated categories of biological processes between two groups. (D) The 15 most significantly enriched KEGG pathways in two groups.

Transcriptomic profiling was conducted to determine the changes in gene expression following LPS injection. After quality filtering, a total of 205,369,969 clean reads were obtained from the LPS group while 196,923,584 high‐quality reads were retained in the control group. Based on differential expression analysis, 79 genes exhibited significant transcriptional changes between the 2 conditions, including 40 upregulated and 39 downregulated genes. The distribution of these DEGs is illustrated in a volcano plot (Figure 4A).

To gain further insight into the biological roles of these DEGs, GO and KEGG enrichment analyses were conducted. The top 10 most significantly enriched GO terms are illustrated in Figure 4B. Within the biological processes category, gene terms associated with the regulation of lipid metabolic process (GO:0019216), regulation of steroid biosynthetic process (GO:0050810), regulation of steroid metabolic process (GO:0019218), and steroid biosynthetic process (GO:0006694) were found to be downregulated in the LPS group (Figure 4C) while gene terms associated with regulation of inflammatory response, regulation of immune effector process and regulation of response to external stimulus were found to be upregulated in LPS group. The most significantly enriched KEGG pathways primarily involved the IL‐17 signaling pathway, Circadian rhythm, and African trypanosomiasis pathways (Figure 4D). qPCR was employed to validate the transcriptomic data. The qPCR results exhibited a strong positive correlation with RNA‐seq measurements, thereby confirming the accuracy and consistency of the sequencing‐based expression profiles (Supporting Information S2: Figure 2).

### 3.4 Correlation Analysis of Metabolome and Transcriptome Under LPS

**Figure 5:**
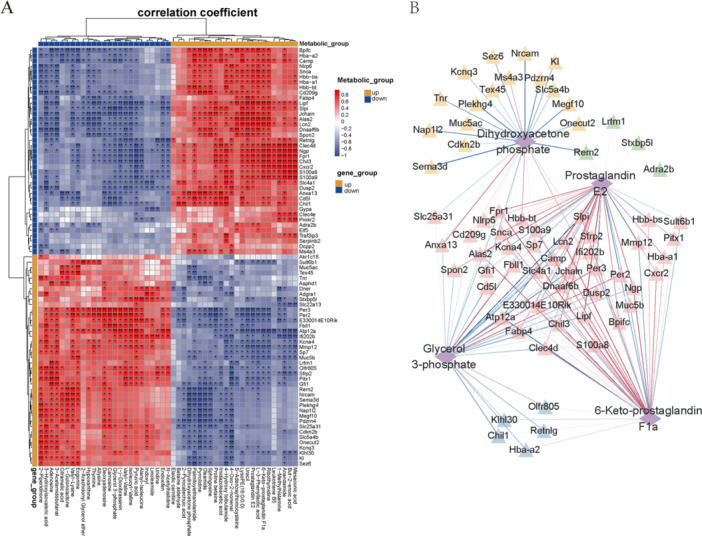
Correlation and pathway analysis of significant differential expressed metabolites and differential expressed genes. (A) Heatmap showed the correlation between metabolites and genes. (B) The plot of targeting relationship network between metabolites and genes. *p < 0.05, **p < 0.01.

**Figure 6:**
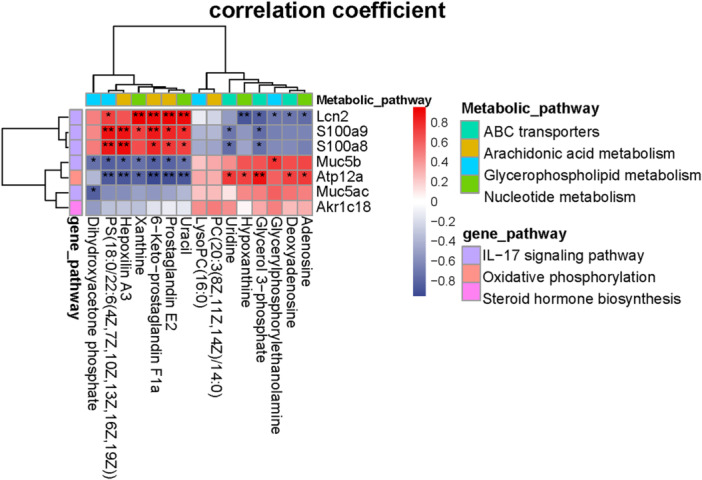
Correlation analysis of significant differential expressed metabolites in four pathways and differential expressed genes in three pathways. *p < 0.05, **p < 0.01.

We used correlation analysis of differential metabolites and transcripts to elucidated the regulatory roles of genes in metabolic pathway in mice ovary under inflammation caused by LPS injection (Figure 5A). The expression level of genes was highly correlated with prostaglandin E2, 6‐keto‐prostaglandin F1a, dihydroxyacetone phosphate, and glycerol 3‐phosphate identified in ovarian tissues (Figure 5B). The IL‐17 signaling and oxidative phosphorylation pathways, which may affect ovarian steroidogenesis, were emphasized. Five genes (Lcn2, S100a9, S100a8, Muc5b, Muc5ac) in IL‐17 signaling pathway were positively related to prostaglandin E2 and 6‐keto‐prostaglandin F1a, which were the catabolites of polyunsaturated fatty acids while negatively related to dihydroxyacetone phosphate, the metabolites related to glycolysis. Atp12a in oxidative phosphorylation pathway was negatively related to prostaglandin E2 and 6‐keto‐prostaglandin F1a while positively related to glycerol 3‐phosphate (Figure 6).

To investigate the PPI networks of the significantly differentially expressed proteins, a functional analysis of 97 such proteins was conducted using the STRING database. KEGG pathway enrichment analysis indicated that a subset of these proteins is associated with the IL‐17 signaling pathway, which may contribute critically to the pathogenesis of low ovarian response induced by LPS exposure (Supporting Information S3: Figure 3).

## 4 Discussion

This study presents evidence that LPS can result in a reduced ovarian response, which was evidenced by a marked reduction in both the quantity and proportion of MII oocytes in the LPS‐treated group. Additionally, our results indicate that LPS impacts the IL‐17 signaling pathway and oxidative phosphorylation pathway, leading to alterations in metabolic processes.

In our study, we observed a decrease in metabolites related to carbohydrates and lipids in ovarian tissue, as well as a decrease in certain metabolites related to amino acids and fatty acids in serum samples in the LPS group. The oocyte is a cell with high energy demands. Any nutritional alterations influencing the energy balance can potentially impact folliculogenesis. The depletion of amino acids, for instance, can disrupt folliculogenesis and oogenesis by impairing the rapamycin signaling pathway [16]. Supplementation with essential amino acids l‐proline and l‐glutamine promotes nuclear maturation of bovine oocytes during in vitro maturation [17]. The balance of free fatty acids, particularly nonesterified fatty acids, plays a crucial role in oocyte development in mammals [18]. In murine models, fatty acid oxidation in the COC serves as a significant energy source for the oocyte, with deficient fatty acid oxidation in vitro recognized as a model of poor oocyte quality [19]. In humans, increased concentrations of free fatty acids can induce lipotoxicity and inhibit oocyte maturation [20]. Indeed, there were variations in metabolomics analysis between ovary and serum samples. Nevertheless, it is noteworthy that the top 5 metabolite categories and the most enriched KEGG pathways showed similarities between serum and ovary tissues. The minor discrepancies observed can be attributed to the fact that metabolites in blood are influenced to a greater extent by recent diet, stress, and nutritional status compared to those in ovarian tissue [21].

Analysis of the transcriptome profile revealed that genes involved in the regulation of lipid metabolic processes were downregulated in the LPS group according to GO analysis. We hypothesized that LPS‐induced alterations in lipid, carbohydrate, and amino acid metabolites may ultimately lead to a reduced response of the ovary to controlled ovarian stimulation.

Infections can present with observable symptoms or remain asymptomatic. Previous studies have shown that even low levels of follicular LPS can disrupt ovarian function. In a murine model, pretreatment with LPS was shown to enhance follicular apoptosis and atresia, accompanied by a diminished progesterone response to follicle‐stimulating hormone (FSH) [22]. In LPS‐treated mice, a significant reduction in the proportion of Graafian follicles and the number of corpora lutea was observed, likely resulting from apoptosis induction and immune cell activation within the ovary, leading to increased production of pro‐inflammatory cytokines [23]. In the pZP3‐induced autoimmune ovarian disease mouse model, which leads to premature ovarian failure, the expression of genes associated with pro‐inflammatory M1 macrophages was significantly upregulated [24]. Subacute infections have the potential to impact the ovarian environment and may be an underappreciated factor contributing to decreased female fertility. This effect could be attributed to the modulation of ovarian E2 signaling in follicular granulosa cells by inflammation, through the NF‐κB and WNT/β‐catenin crosstalk [25].

In our study, transcriptomic analysis revealed that the most significantly enriched KEGG pathways included the IL‐17 signaling pathway, suggesting that IL‐17 may play a role in this pathological process. The IL‐17 family members play a role in defending against extracellular fungal and bacterial pathogens, as well as in certain immune‐pathologies such as autoimmune diseases and cancer progression [26, 27]. IL‐17 activation induces NF‐κB expression [28], which in turn amplifies inflammation. Our study demonstrated that the IL‐17 signaling pathway is positively associated with catabolites of polyunsaturated fatty acids and negatively associated with metabolites related to glycolysis. This may represent a potential mechanism underlying the impact of LPS on ovarian response.

In this study, we employed an integrated transcriptomic and metabolomic analysis strategy. By concurrently analyzing both serum and ovarian tissue samples from mice, we elucidated the effects of LPS on ovarian superovulation responsiveness. Our findings contribute novel insights into the potential molecular mechanisms underlying POR, thereby broadening the current understanding of its pathophysiology. There were some limitations in our study. Although we identified an association between LPS exposure and impaired ovarian responsiveness, the precise molecular mechanisms driving this relationship remain unclear. Future investigations will focus on elucidating the underlying regulatory pathways, particularly those involved in metabolic processes, to provide a more comprehensive mechanistic understanding.

In conclusion, our study provided evidence of inflammation on damaging ovary response to controlled ovarian stimulation, linked to dysfunctions in lipid, carbohydrate, and amino acid metabolism. Integrative analysis of transcriptomic and metabolic showed IL‐17 signal pathways may play a critical role in this process. In future studies, we aim to increase the sample size and conduct more comprehensive mechanistic investigations to further substantiate our findings.

## Author Contributions

Xin Mu and Mei‐Li Pei jointly drafted and finalized the manuscript based on feedback from all co‐authors. Feng Zhu participated in project design and critically revised the manuscript. Yudan Zhang performed and validated all statistical analyses involved in this study. Mei‐Li Pei is responsible for the conceptualization of the study. All authors reviewed the manuscript, contributed to the interpretation of its intellectual content, and approved the final version for publication.

## Ethics Statement

This study received approval by the Health Science Center of XJTU Approval for Research Involving Animals (XJTUAE2024‐1872).

## Consent

The authors have nothing to report.

## Conflicts of Interest

The authors declare no conflicts of interest.

## Supporting information


**Supporting Figure 1:** Concentration of interleukin‐17 (IL‐17) between two groups.

**Supporting Figure 2:** Correlation and Histogram analysis of differentially expressed genes obtained from RNA‐seq and qPCR. (A) Correlation analysis of differentially expressed genes between RNA‐seq and qPCR; (B) Histogram analysis of differentially expressed genes between RNA‐seq and qPCR.

**Supporting Figure 3:** (A) Protein–protein interaction (PPI) networks based on significant differentially expressed proteins; (B) KEGG pathway analysis of significant differentially expressed proteins.

**Supplementary table 1:** Information of primer sequences in qPCR.

## Data Availability

The data sets analyzed during the current study are available from the corresponding author on reasonable request.
