# Supplementary figures and images for: Integration Analysis of Metabolome and Transcriptome Reveals the Effect of Lipopolysaccharide on Ovary Response to Stimulation
Source: Immun Inflamm Dis. 2025 Dec 24;13(12):e70309. doi: 10.1002/iid3.70309 (PMC12728487; doi:10.1002/iid3.70309)

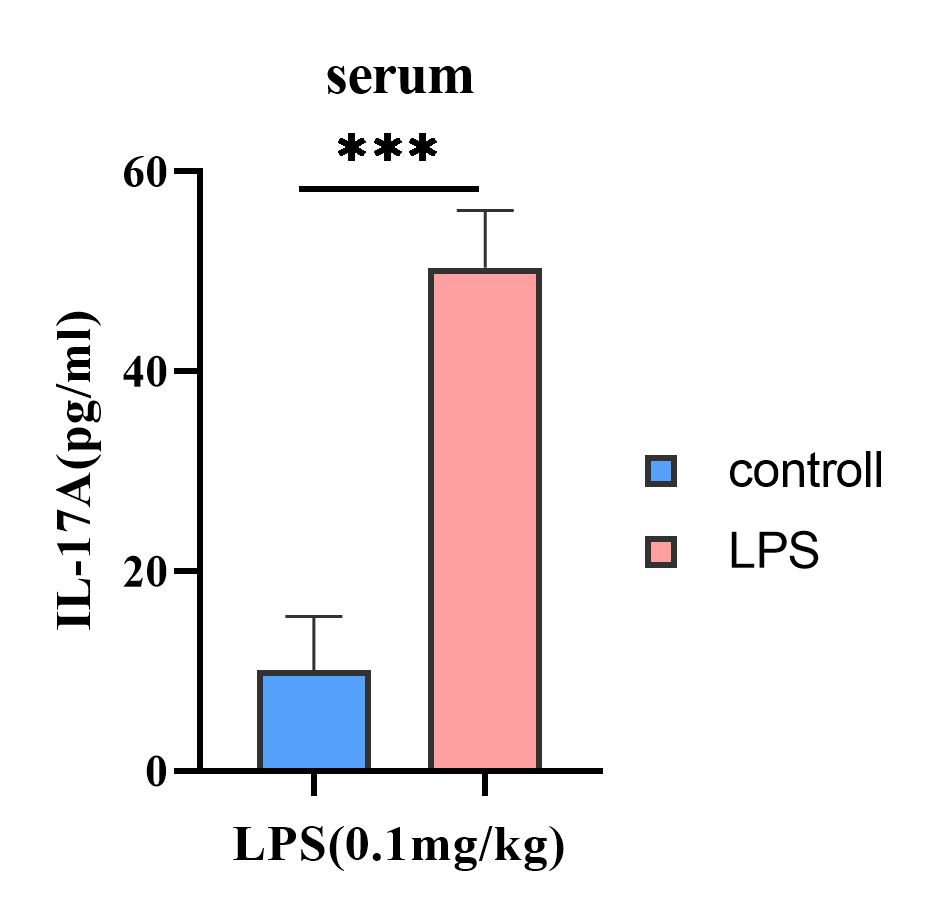

Supplement: Supplementary file 1 — Supporting Figure 1: Concentration of interleukin‐17 (IL‐17) between two groups. [file IID3-13-e70309-s001.png]

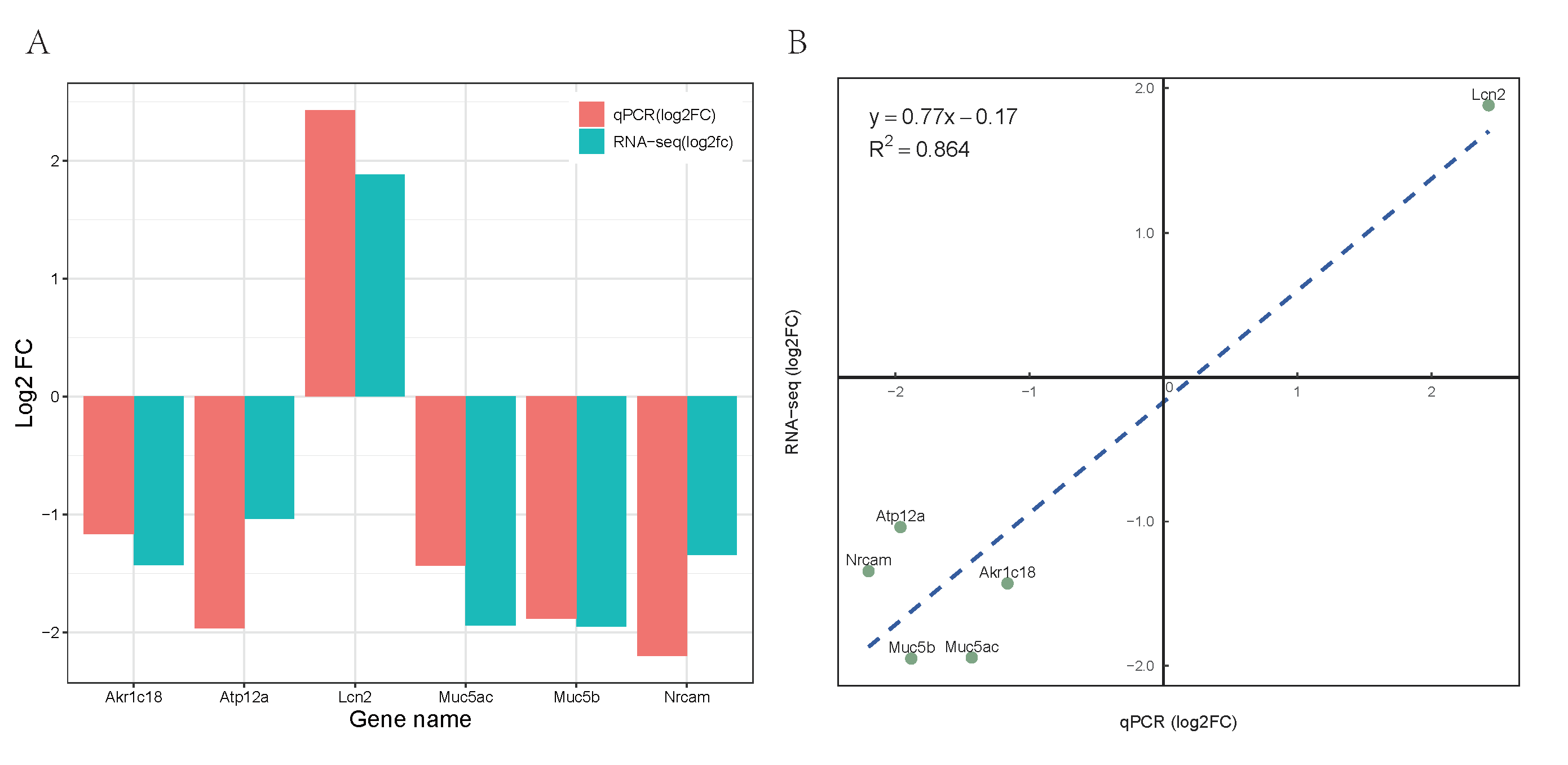

Supplement: Supplementary file 2 — Supporting Figure 2: Correlation and Histogram analysis of differentially expressed genes obtained from RNA‐seq and qPCR. (A) Correlation analysis of differentially expressed genes between RNA‐seq and qPCR; (B) Histogram analysis of differentially expressed genes between RNA‐seq and qPCR. [file IID3-13-e70309-s004.png]

A

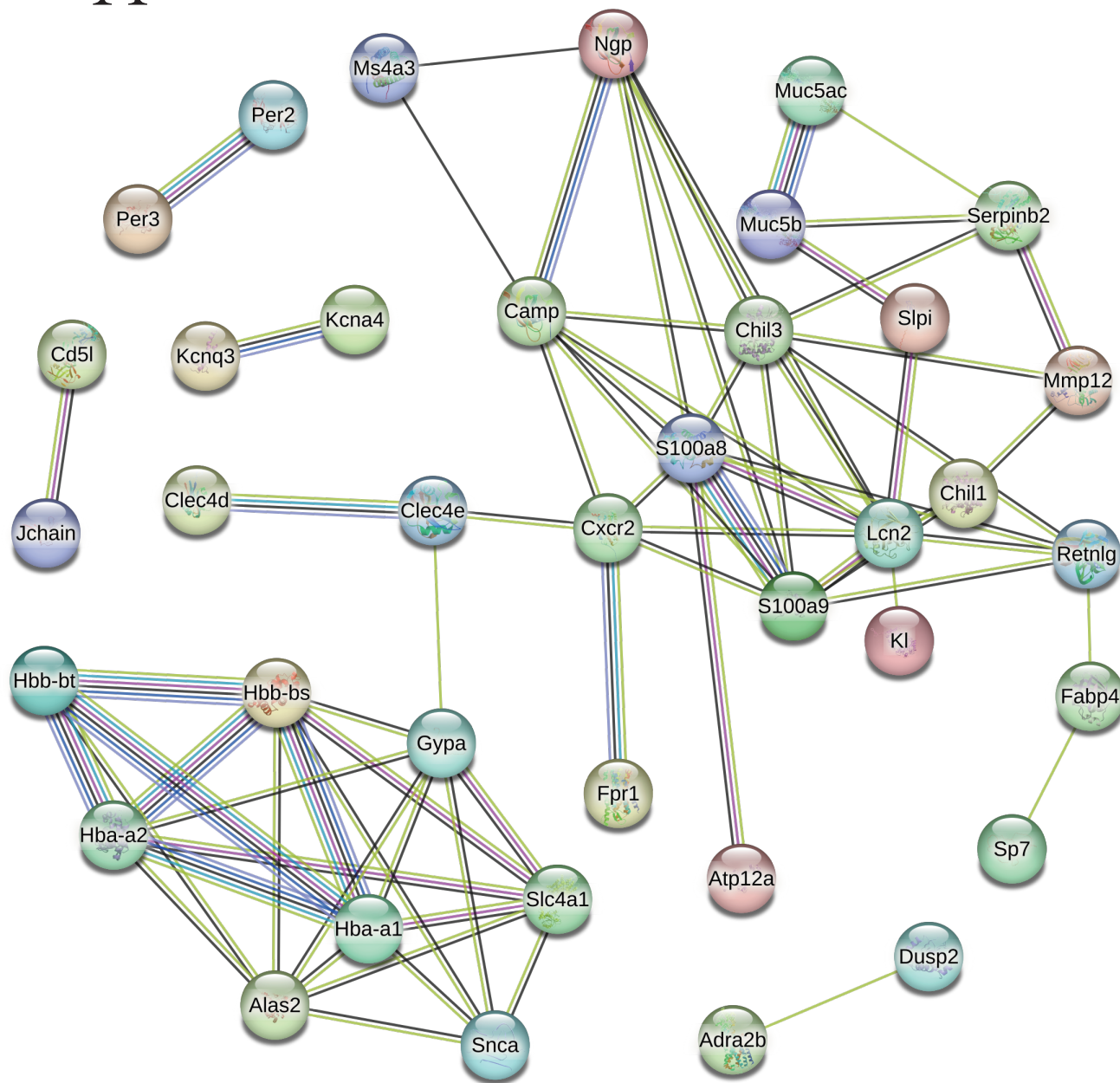

B

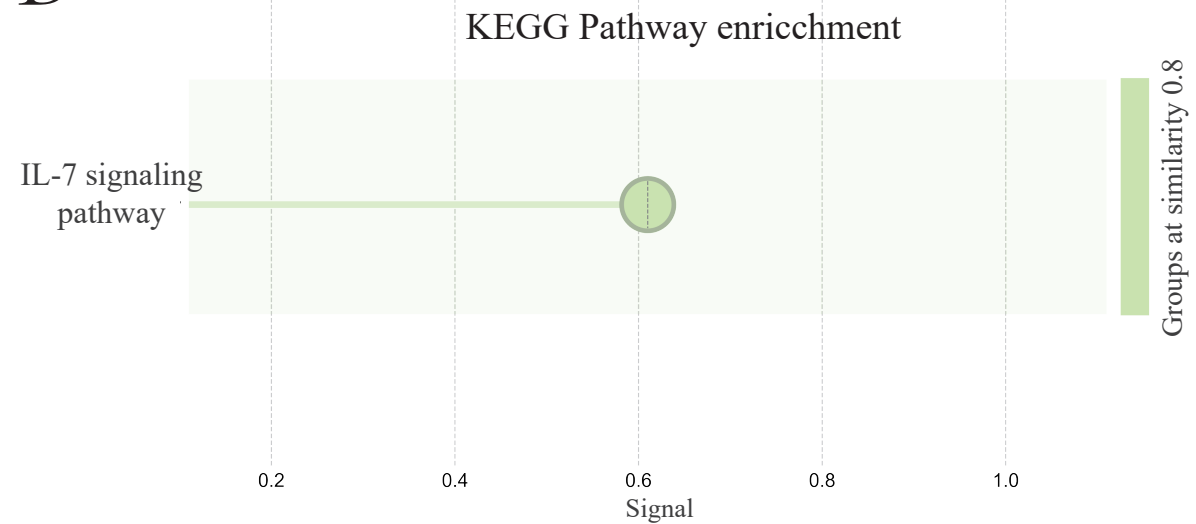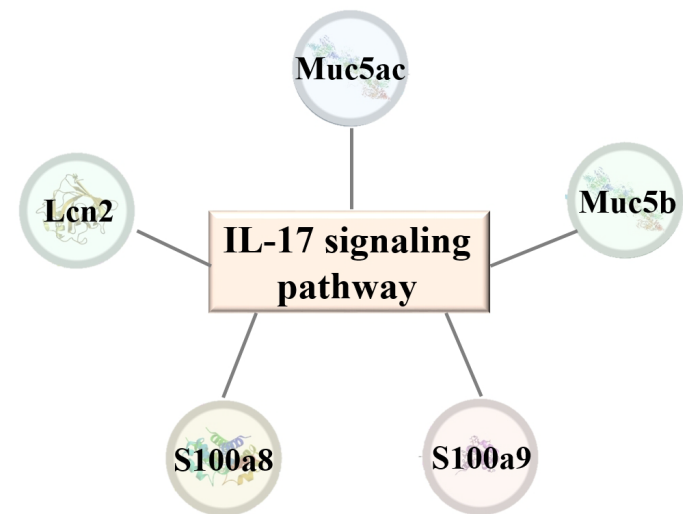

Supplement: Supplementary file 3 — Supporting Figure 3: (A) Protein–protein interaction (PPI) networks based on significant differentially expressed proteins; (B) KEGG pathway analysis of significant differentially expressed proteins. [file IID3-13-e70309-s002.pdf]
