# Supplementary material for: Integration Analysis of Metabolome and Transcriptome Reveals the Effect of Lipopolysaccharide on Ovary Response to Stimulation
Source: Immun Inflamm Dis. 2025 Dec 24;13(12):e70309. doi: 10.1002/iid3.70309 (PMC12728487; doi:10.1002/iid3.70309)
Supplement: Supplementary file 4 — Supplementary table 1: Information of primer sequences in qPCR. [file IID3-13-e70309-s003.docx]

Supplementary table 1. Information of primer sequences in qPCR

| Genes | Sequences (5′-3′)  F: Forward; R: Reverse | Accession No. | Length (bp) |
| --- | --- | --- | --- |
| MUC5b | F: ATCCGCCTAGTCCTCACCTT  R: TTTCCAAACTGCACAGGGGT | NM_028801.2 | 170 |
| ATP12a | F: GCGTGTGTTGGGTTTCTGTC R：AGACCGAGGAGGGTCAATCA | NM_138652.2 | 142 |
| AKR1C18 | F: ATTCCTGTCCTGGGCTTTGG  R: TGGCCTGGCCTATCTCTTCT | NM_001346535.1 | 148 |
| Muc5ac | F：TGTGGTACGAGCCTTCAACC  R：TCCTCATAGGCATCCCCACA | NM_010844.3 | 150 |
| LCN2 | F：GGACTACAACCAGTTCGCCA  R：CCTTGAGGCCCAGAGACTTG | NM_008491.2 | 149 |
| NRCAM | F：AAGGACAATGGAGAGCTGCC R：CACTGTCCAGCGTTGTGTTG | XM_036157483.1 | 127 |
